# Genetic variation in the Solanaceae fruit bearing species lulo and tree tomato revealed by Conserved Ortholog (COSII) markers

**DOI:** 10.1590/S1415-47572010005000016

**Published:** 2010-06-01

**Authors:** Felix Enciso-Rodríguez, Rodrigo Martínez, Mario Lobo, Luz Stella Barrero

**Affiliations:** 1 Biotechnology and Bioindustry Center, Colombian Corporation for Agricultural Research, CORPOICA, Mosquera, CundinamarcaColombia; 2 La Selva Research Center, CORPOICA, Rionegro, AntioquiaColombia

**Keywords:** COSII, genetic diversity, lulo, tree tomato

## Abstract

The Lulo or naranjilla (*Solanum quitoense* Lam.) and the tree tomato or tamarillo (*Solanum betaceum* Cav. Sendt.) are both Andean tropical fruit species with high nutritional value and the potential for becoming premium products in local and export markets. Herein, we present a report on the genetic characterization of 62 accessions of lulos (n = 32) and tree tomatoes (n = 30) through the use of PCR-based markers developed from single-copy conserved orthologous genes (COSII) in other Solanaceae (Asterid) species. We successfully PCR amplified a set of these markers for lulos (34 out of 46 initially tested) and tree tomatoes (26 out of 41) for molecular studies. Six polymorphic COSII markers were found in lulo with a total of 47 alleles and five polymorphic markers in tree tomato with a total of 39 alleles in the two populations. Further genetic analyses indicated a high population structure (with F_ST_ > 0.90), which may be a result of low migration between populations, adaptation to various niches and the number of markers evaluated. We propose COSII markers as sound tools for molecular studies, conservation and the breeding of these two fruit species.

## Introduction

The Lulo or naranjilla (*Solanum quitoense* Lam.) and the tree tomato or tamarillo (*Solanum betaceum* Cav. Sendt.) are edible fruits belonging to the Solanaceae family. The primary center of diversity for lulo comprises the Andean region of Colombia, Ecuador and Peru (Whalen *et al.*, 1981; Heiser and Anderson, 1999; Lobo *et al.*, 2007), whereas that for the tree tomato, besides the aforementioned, also includes Bolivia (Bohs, 1994). These fruits are mainly planted in the Andean region, expanding there from to other parts of the world, lulos to the subtropics of Central America and tree tomatoes to New Zealand and various Asian and African countries. The fruit of both can be eaten either raw or cooked, besides being widely used for making juice, pies, jelly, jam, ice cream, as well as for medicinal purposes. In addition, they are noted for their nutritional value due to the high content of vitamins A and C, minerals and carbohydrates (Heiser and Anderson, 1999; Agronet, 2009).

Based on a series of controlled crosses, phylogenetic relationships in lulos, tree tomatoes and related species have been established. *S. quitoense* can form fertile hybrids with *S. hirtum*, whereas in *S. betaceum*, this is possible with *S. unilobum* (Heiser, 1972; Bohs, 1991, 1994; Bernal *et al.*, 1998 Lobo *et al.*, 2000, 2007). These findings have been useful for generating improved material, thereby culminating in a cultivar known as “Lulo La Selva”, a cross between *S. quitoense* and *S. hirtum*, which is now available to the public (Bernal *et al.*, 1998). As regards tree tomatoes, no improved breeding material is publicly available. Nevertheless, as a result of breeding programs, various hybrids between *S*. *betaceum* and *S*. *unilobum* are being evaluated in Colombian fields (Lobo *et al.*, 2000).

In order to further genetic improvement, the characterization of a broad genetic base is required. In accordance, the Colombian Corporation for Agricultural Research -CORPOICA- maintains 159 entries of lulos and related species from the Lasiocarpa section, and 75 tree tomatoes and related taxa from Ecuador, Peru, Bolivia, Brazil, Venezuela, Costa Rica, Trinidad and India, together with a large representation from Colombia itself. These collections have been partially characterized by using phenotypic information (Benitez *et al.*, 1991; Lobo *et al.*, 2007). Furthermore, the lulo collection has been additionally characterized through molecular AFLP markers (M. Lobo, unpublished results). Nevertheless, additional informative (co-dominant) markers are necessary for a precise assessment of genetic variation, for use in both conservation and breeding programs.

In 2004, a consortium of some 30 countries embarked on an International Solanaceae Genomics Project -SOL- , with the mission of developing genomics tools for this family (Mueller *et al.*, 2005). One of the tools developed by SOL is the Conserved Ortholog Set (COS) markers, suitable for evolutionary, phylogenetic, and comparative genomic studies in Asterid species where the Solanaceae family is widely represented (Fulton *et al.*, 2002). The comparison of tomato ESTs to pepper, potatoes, eggplants, coffee and the complete gene set from *Arabidopsis*, has resulted in a combined COS set composed of 2,869 unigenes across these species. This second generation of markers (COSII) includes *Universal Primers for Asterid species* (UPA) from COS genes designed to amplify intronic (iUPA) and/or exonic (eUPA) regions, thereby providing greater flexibility in the identification of polymorphisms (Wu *et al.*, 2006). More recently, additional sets of COS genes have been generated for diversity studies in tomatoes (Van Deynze *et al.*, 2007). Due to their conserved nature, COSII markers may be transferred among species of the Asterid clade through amplification of universal primers without the need for investing in sequencing costs, thus representing an economic alternative to other types of co-dominant markers such as SSRs, whose generation is usually costly and time-consuming, as prior sequencing steps are required for their development. We evaluated COSII markers in lulos and tree tomatoes in a sample from the CORPOICA collection, and successfully transferred markers for studies of genetic variation in these species. Here, we show how genomic information maintained by the SOL project in well-known species, such as tomatoes, can be used in species where little or no genomic information is available, such as lulos and tree tomatoes.

## Materials and Methods

###  Plant material and DNA isolation

Thirty accessions of lulos, two entries of the related species *S. hirtum*, and 26 accessions of tree tomatoes, as well as four entries of the relative species *S. corymbiflora*, *S. diversifolia* and *S. hartwegii* (Table 1), with five seedlings per accession, were selected from the CORPOICA germplasm collection based on geographic distribution. Genomic DNA was isolated from young leaves of each seedling, following the procedure described by Fulton *et al.* (1995), by using approximately 2 g of leaves in 1 mL of extraction buffer, with the exception of the final concentration of sodium bisulfite, which was 1.6% (w/v). The quantity and quality of genomic DNA were checked using a Beckman DU^®^ 530 spectrophotometer and on 1% (w/v) agarose gels.

###  PCR amplification

Forty six candidate COSII markers for lulos and 41 for tree tomatoes, were selected mainly *in silico* from more than 400 markers, based on the presence of Single Nucleotide Polymorphisms (SNPs) or Insertions/Deletions (InDels) in the lulos and tree tomatoes themselves (Table *S1*; Pratt *et al.*, 2008). PCR amplification was carried out in an i-Cycler thermal Cycler (Bio-Rad, Hercules, CA, USA) as follows: one cycle of initial denaturation for 5 min at 94 °C, followed by 35 cycles for 30 s at 94 °C, 1 min at 55 °C and 2 min at 72 °C, followed by a final extension of 10 min at 72 °C, and preservation at 4 °C until further analysis. PCR conditions were optimized for 25 μL of reaction mixture by using 0.1 μM of each primer and 25 ng of template DNA. Amplification products were separated by gel electrophoresis on 2% (w/v) agarose in a 1X TAE buffer (40 mM Tris-acetate and 1 mM EDTA), and then stained with ethidium bromide (0.5 μg/mL).

###  Statistical analysis

Stained PCR products were visualized with GeneSnap software for Windows XP, using the 1 kb plus DNA ladder as standard (Invitrogen, Carlsbad, CA, USA). Products showing different sizes (pb) *i.e.* polymorphic by insertions/deletions (InDels > 20 pb) were considered different alleles of the same COSII locus. The number of alleles per COSII locus was counted, whereupon each allele was assigned a consecutive number. A matrix of alleles per locus *vs.* accessions was generated.

Ho (observed heterozygosity) and He (expected heterozygosity) were calculated as described by Hartl (1987). Population structure was estimated using Wright's F-statistics (F_IS_: inbreeding coefficient, F_IT_: measure of the genetic differentiation over populations, F_ST_: variance of allele frequencies among populations) (Wright, 1965).

Principal component (PC), genetic diversity, and population structure analysis were performed using Genetix 4.05 (Belkhir *et al.*, 2004). Genetic distances were calculated using the Cavalli-Sforza distance measure (Cavalli-Sforza and Edwards, 1967), as this distance-method is employed to analyze gene frequencies without complying to any biological assumption, and is especially useful in cases where little is known about the evolutionary forces driving genetic change. Dendograms were generated by way of the Unweighted Pair Group Method (UPGMA). Bootstrapping with 10,000 replicates was carried out to assess statistical support for each cluster. Consensus trees were constructed using PHYLIP 3.5 (Felsenstein, 1989).

## Results and Discussion

###  Polymorphism

Polymorphism assessed in lulos showed that among 46 COSII markers initially selected, 34 were successfully transferred and PCR amplified, six being polymorphic by InDel as observed by agarose gel electrophoresis. The polymorphic COSII markers were previously classified as iUPAs due to amplification of intronic regions and also mapped to four different tomato chromosomes (Mueller *et al.*, 2005; Table 2). The six COSII markers tested on the panel of 32 accessions revealed a total of 47 alleles and an average of 7.8 alleles per locus. The locus C2_At4g37280 presented the highest number of alleles (15) and the locus C2_ At4g38810 the lowest (four) (Table 2).

In tree tomatoes, among the 41 initially selected COSII markers, 26 were successfully transferred, five of these being polymorphic by InDel as observed by agarose gel electrophoresis. The polymorphic COSII markers were mapped to five different tomato chromosomes and classified as iUPAs, with the exception of locus C2_At3g15430, which was identified as eUPA, due to amplification of the exonic region (Mueller *et al.*, 2005). The five COSII markers tested on a panel of 30 accessions revealed a total of 39 alleles and an average of 7.8 alleles per locus. The number of alleles was the highest (9) in locus C2_4g32930, and the lowest (6) in C2_At3g15430 (Table 2).

To our knowledge, this is the first report describing the use of Asterid COSII polymorphisms for studies on diversity, based on the size of PCR products. However, it is possible that genetic diversity is thereby underestimated, due to the low resolution provided by 2% agarose gels, since differences in a few base pairs (less than 20) are not detected on analysis. Other reports are based on COS sequence information, probably more informative and reliable than that founded on PCR product size (Nakitandwe *et al.*, 2007; Van Deynze *et al.*, 2007). Nevertheless, the evaluation of InDels (> 20 base pairs) in agarose gels could provide valuable information and represent an economic alternative to direct Sanger DNA sequencing. Further studies of polymorphisms based on the estimation of PCR size on gel and the sequencing of PCR products by using the same COSII, are imperative for comparison.

We compared the results generated in this study based on PCR product size using COSII, with others employing microsatellites (SSRs) in Asterids. We found that the number of COSII polymorphic markers identified herein (5-6 polymorphic out of 26-34 initially tested, about 19% and 18%, respectively, Table 2) were comparable to reports on results with SSRs, despite fundamental differences in the markers used to estimate genetic diversity, nevertheless, both markers are similar as regards the presence of multiple and null alleles, co-dominance and presence of InDels. For example, in potatoes, three SSR markers were polymorphic out of 27 initially tested (about 11%, Bornet *et al.*, 2002), and, 18 out of 156 were polymorphic in the same species (about 12%, Ghislain *et al.*, 2004). Likewise, the average number of alleles per locus was higher in the present study (7.8 per species) when compared to other SSR reports. For example, in diploid coffee, four alleles per locus were found (Moncada and McCouch, 2004), and in tomatoes, 2.7 (He *et al.*, 2003). Since these comparisons are among different species, material and mating systems, care must be taken and the results only considered indicative of successful COSII rates. Further comparison with SSRs within the same entries and species evaluated is desirable.

###  Genetic diversity and population structure

Heterozygosity values per accession and locus were low, with Ho and He averages of 0.03 and 0.05 for lulos and 0.003 for tree tomatoes (Table 3). A preliminary population structure analysis, with the data assessed both per accession and locus, revealed extremely high values. Thus, F_IS_, F_IT_, and F_ST_ averages per accession were 0.42, 0.96, 0.93 for lulos, and 0.86, 0.99, 0.99 for tree tomatoes, respectively. Averages per locus were 0.47, 0.96, 0.92 for lulos, and 0.43, 0.99, 0.99 for tree tomatoes, respectively (Table 3). The high F_IS_ and F_IT_ values are related to a deficit in heterozygotes within accessions (subpopulations) and are in accordance with the low Ho and He values per accession and locus (Table 3). Analysis revealed population structures in both lulos and tree tomatoes, with high homozygosis (fixation of alleles) within accessions and high mutual genetic differentiation, *i.e.* almost no sharing of alleles.

The high population structure and diversity may be explained by several factors including, in the first place, geographical distribution. Both lulos and tree tomatoes are natives of the Andean region, near the equator, comprising dramatically variable habitats, as rain forests, deserts and high mountains, with regular snowfall and sub-freezing temperatures. Thus the species were unperturbed by ice age conditions, and may have had time to accumulate adaptive genetic variation to extreme ecological niches (Heiser and Anderson, 1999; Bohs, 2004; Mueller *et al.*, 2005; Lobo *et al.*, 2007). In the present study, we used a different geographical sample representing several Colombian regions (Table 1), this including a wide range of habitats ideal for the establishment of different niches, possibly inapt for genetic flow among accessions. In the second place, sampling populations were obtained mainly from small orchards planted by local farmers with few individuals per location, and with the absence of or reduced migration between demes, thus probably giving rise to low sharing of alleles among accessions and genetic drift. The third point is the mating system. Although some studies have reported a high natural cross pollination rate, both lulos and tree tomatoes have also been described as self-compatible species (Heiser, 1972; Benítez *et al.*, 1991; Bohs, 1994), thus adept to self-fertilization, propitious for the fixation of alleles. Finally, the type of markers, as the only type of COSII polymorphism analyzed in this study were insertions / deletions (InDels > 20 base pairs), apparent in the different sizes of PCR products. We could also consider as another type of polymorphism, the presence of null alleles, which may contribute to the shortage of heterozygotes observed within populations, a phenomenon also reported for SSRs (Varshney *et al.*, 2005).

###  Principal component and cluster analysis

**Figure 1:**
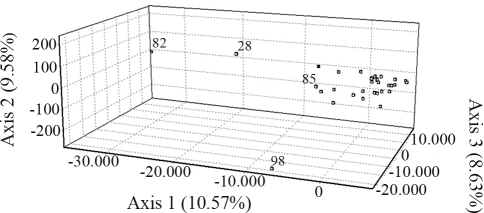
PCA analysis for lulos. Only accessions differing from the main constellation are represented by their last two numbers.

**Figure 2:**
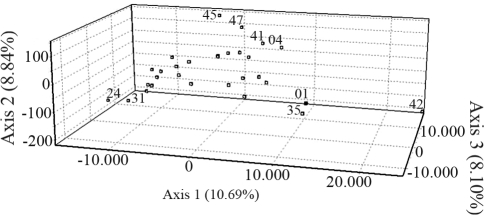
PCA analysis for tree tomatoes. Only accessions differing from the main constellation are represented by their last two numbers.

PC analysis revealed four factors (axes) explaining 36.82% of the total genetic variation in lulos and 35.27% in tree tomatoes, each factor contributing a similar percentage of variance (8.04-10.57% for lulos and 7.65-10.69% for tree tomatoes). The distribution of populations in the three dimensions revealed by the first three PCs showed defined groups (Figures 1  and 2). In lulos, most accessions formed a group at the first axes, although accession 05T1688028 was far from the centroid of the three axes. Accession 06L082 was found far from the centroid of the first and third axis. Finally, accessions 06L098 and 06L085 were different from the other accessions at the third axis (Figure 1). In tree tomatoes, accession 06TA042 was far from the centroid of the three axes. Accessions 06TA024 and 06TA031 were far from the centroid of the first and second axis, whereas 06TA041, 06TA045 06TA004 and 06TA047 were far from the centroid of the second axis. Finally, accessions 06TA001 and 06TA035 were found to be to the right of the center of the first axis (Figure 2).

To estimate genetic distances, we used the Cavalli-Sforza distance measure (Cavalli-Sforza and Edwards, 1967). The consensus UPGMA tree per species, derived from genetic distances (Tables *S2* and *S3*), displayed clusters with low to high statistical support, with bootstrapping values ranging from 0.02%-100% for lulos and 0.004%-100% for tree tomatoes. In both cases, accessions 05T1688028 for lulo and 06TA042 for tree tomatoes were chosen as outgroups, with 100% bootstrapping values clustering out of the other accessions (Figures *S1* and *S2*). In lulos, distances were the highest and clusters independent in accessions 06L082, 06L098 and 06L085, although bootstrapping values were low (17%-33%, Figure *S1*). In tree tomatoes, high distances and independent clusters with low bootstrapping support prevailed in accessions 06TA031, 06TA24, 06TA045 and 06TA41 (0.6%-35%, Figure *S2*). These results are consistent with PCA output. Moreover, the low bootstrapping values observed might be related to high genetic differentiation possibly arising from high homozygosity within accessions and the low number of mutually shared alleles, propitious for the formation of several cluster possibilities, each with low statistical support.

*S. hirtum* 05T1688028 (a wild relative to *S. quitoense*) and *S. corymbiflora* 06TA042 (a wild relative to *S. betaceum*) were chosen as outgroups, since PCA output showed these accessions to be consistently and tri-dimensionally separate from the others. This is due to their both coming from different countries as compared to the majority of the accessions tested (05T1688028 was from Venezuela and 06TA042 from Brazil) (Table 1). This separation is also consistent with the two species being distantly related to *S. quitoense* and *S. betaceum* (Heiser, 1972; Bohs, 1994). *S. corymbiflora* is widespread in humid forests of southeastern Brazil and the adjacent Argentina, and is not interfertile with *S. betaceum* (Bohs, 1994). *S. hirtum* grows throughout the tropics and is found near to related species as *S. quitoense* (Heiser, 1972). *S. hirtum* and *S. corymbilora* were under-represented in the tested sample, with only two accessions in the former and one in the latter (Table 1).

The fact that the other *S. hirtum* accession from Santander, Colombia (06L063) grouped distantly from *S. hirtum* from Venezuela (05T1688028) might be consistent with their different geographic origins. Nevertheless, *S. hirtum* 06L063 did group together with other *S. quitoense* accessions, thus possibly indicating allelic homoplasy (the close relationship between species), which may have occurred through the same-sized alleles on agarose not necessarily being homologs, consequently with different and recent common ancestors. Thus, differences in sequence, if any, could be masked as the alleles were of the same size. The same principle might also apply to the close relationships between *S. betaceum, S. hartwegii* var *racemosa*, and *S. diversifolia*, species which grouped together. We also observed a lack of clustering associated with geographic origin in Colombian regions, as well as any other attribute. This might reflect the lack of specific sampling for different geographic and distribution regions. Similar results were obtained by Lobo *et al.* (2007) in a morphological characterization study which included material from the current research.

The pronounced variability observed among lulo accessions is in agreement with previous studies based on morphologic and agronomic traits, thereby indicating the high variation among accessions, most of which represented Colombian entries (Lobo *et al.*, 2007), although this is not the case in *S. quitoense*, where genetic variation is low, based on the morphology of entries from Ecuador (Whalen *et al.*, 1981). This contradiction has been explained by M. Lobo (unpublished results) and Lobo *et al.* (2007), who proposed that Colombia is the possible center of origin of lulo when based on genetic and linguistic premises, *i.e.* most Colombian entries present nondomesticated traits such as the presence of spines, and the name “lulo”, as used in Colombia, is of Quechua (indigenous) origin, whereas the name “naranjilla”, used in Ecuador, is of Spanish origin. Furthermore, these authors, as well as Heiser (1969), mentioned a possible founder effect associated with human dispersion from a common center, namely Colombia.

###  Implications of diversity, conservation, and breeding

Because of their codominant, bi (SNPs -in the case of diploid species) or multi-allelic (InDels) nature, we may infer that COSIIs are adequate for diversity analysis. Due to the novel nature of using COSIIs based on the size of PCR products for diversity, we resorted to a small marker sample, which might thus bias analysis. Consequently, care should be taken in interpretation, with confirmation by means of a larger sample of polymorphic markers. Notwithstanding, this study represents an important guiding line for future research.

COSII markers are being used for studies on diversity based on sequence data in tomatoes (Nakitandwe *et al.*, 2007; Van Deynze *et al.*, 2007). COSII sequence data have been instrumental in arriving at a clear differentiation of population level distribution of genetic variation. Thus, most genetic variation among samples was observed in primary centers of diversity and the least in secondary. Moreover, when compared to other SNP and InDel markers, both non-coding arbitrary and EST-based, COSIIs furnished similar estimates of polymorphism (Labate *et al.*, 2009). Future studies on diversity in lulos and tree tomatoes should include comparisons with COSIIs based on sequence data or other co-dominant markers such as SSRs.

Based on the high genetic differentiation among lulo and tree tomato accessions, we might venture upon some inferences. Firstly, Colombia could be the primary center of diversity as has already been suggested (Heiser, 1969; Heiser and Anderson, 1999; Lobo *et al.*, 2007). This hypothesis should be tested by surveying samples of comparable sizes from outside this region. In the second place, there was usually no mutual difference among individuals within accessions, thereby implying that collection-sampling should be more directed to several populations, to so sample genetic variation to a greater extent. Larger populations and loci sampling should be considered in future studies to confirm the very high population structure observed in this study. Third, success in obtaining hybrid vigor is highly probable by crossing different accessions and species, providing they are genetically distant, whence, high pathogen resistance and yield-heterosis has been observed in various crosses between *S. hirtum* and *S. quitoense* (Heiser, 1972; Bernal *et al.*, 1998), as well as between *S. betaceum* and *S. unilobum* accessions (Lobo *et al.*, 2000)*.* Finally, there should be a high probability of success for gene-map construction and QTL analysis among accessions. Hence, we have observed high levels of COSII polymorphism distributed among the 12 tomato chromosomes in different lulo and tree tomato parental combinations (Pratt *et al.*, 2008).

Finally, we illustrate the potential of the SOL genomics initiative, wherefore the use of COSII markers is just one example of the transferability of genomics tools to less developed Solanaceae species such as lulos and tree tomatoes for their future conservation and breeding.

## Supplementary Material

The following online material is available for this article:

Figure S1 Consensus tree using bootstrapping of 10,000 replicates for lulo.

Figure S2 Consensus tree using bootstrapping of 10,000 replicates for tree tomato.

Table S1 COSII Markers selected for screening.

Table S3 Genetic distances for tree tomato and relatives.

This material is available as part of the online article from http://www.scielo.br/gmb

## Figures and Tables

**Table 1:** Accessions of lulos, tree tomatoes and the related taxa used in this study.

Species	Accession	Origin^1^	Species	Accession	Origin^1^
*S. quitoense*	04T14203	Valle	*S. betaceum*	06TA001	Antioquia
*S. quitoense*	06L053	Huila	*S. betaceum*	06TA004	Nariño
*S. quitoense*	06L054	Peru*	*S. betaceum*	06TA007	Nariño
*S. quitoense*	06L061	Antioquia	*S. betaceum*	06TA008	Cauca
*S. quitoense*	06L062	Antioquia	*S. betaceum*	06TA009	Valle
*S. quitoense*	06L064	Putumayo	*S. betaceum*	06TA010	Antioquia
*S. quitoense*	06L066	Valle	*S. betaceum*	06TA011	Caldas
*S. quitoense*	06L068	Nariño	*S. betaceum*	06TA013	Caldas
*S. quitoense*	06L069	Valle	*S. betaceum*	06TA014	Boyaca
*S. quitoense*	06L070	Cauca	*S. betaceum*	06TA015	Tolima
*S. quitoense*	06L071	Cauca	*S. betaceum*	06TA016	Tolima
*S. quitoense*	06L072	Valle	*S. betaceum*	06TA018	Antioquia
*S. quitoense*	06L074	Cauca	*S. betaceum*	06TA020	Antioquia
*S. quitoense*	06L075	Cauca	*S. betaceum*	06TA022	Unknown
*S. quitoense*	06L078	Valle	*S. betaceum*	06TA023	Tolima
*S. quitoense*	06L080	Valle	*S. betaceum*	06TA024	Antioquia
*S. quitoense*	06L081	Valle	*S. betaceum*	06TA025	Narino
*S. quitoense*	06L082	Valle	*S. betaceum*	06TA026	Huila
*S. quitoense*	06L084	Unknown	*S. betaceum*	06TA028	Antioquia
*S. quitoense*	06L085	Magdalena	*S. betaceum*	06TA031	Antioquia
*S. quitoense*	06L088	Antioquia	*S. betaceum*	06TA032	Antioquia
*S. quitoense*	06L089	Unknown	*S. betaceum*	06TA033	Antioquia
*S. quitoense*	06L090	Antioquia	*S. betaceum*	06TA034	Nariño
*S. quitoense*	06L091	Costa Rica*	*S. betaceum*	06TA039	Kenya*
*S. quitoense*	06L093	Boyaca	*S. betaceum*	06TA041	Ecuador*
*S. quitoense*	06L094	Antioquia	*S. betaceum*	06TA047	Caldas
*S. quitoense*	06L095	Magdalena	*S. corymbiflora*^2^	06TA042	Brazil*
*S. quitoense*	06L097	Santander	*S. diversifolia*^2^	06TA050	Unknown
*S. quitoense*	06L098	Antioquia	*S. diversifolia*^2^	06TA045	Venezuela*
*S. quitoense*	06L099	Norte de Santander	*S. hartwegii* var*. racemosa*^2^	06TA035	Tolima
*S. hirtum*^2^	05T1688028	Venezuela*			
*S. hirtum*^2^	06L063	Santander			

^1^The region of original sampling is indicated for accessions from Colombia. ^2^Related taxa. Accessions from other countries are denoted by *. All accessions are conserved as seeds.

**Table 2:** Polymorphic loci for lulos, tree tomatoes and relatives.

Species	Locus	Functional annotation^1^	Primer sequence (5'-3')^2^	Tomato chromosome^3^	Number of alleles	Size range of PCR product^4^ (bp^5^)
Lulo and relatives	C2_At5g66530	Aldose 1-epimerase family protein	F-TTCAGGAATGGCATTTGCAAGTGTG R-ACCATTGAATACAGCATCTGGTCGAAC	2	10	743-1594
	C2_At2g34470	Encodes a urease accessory protein, essential for the activation of plant urease	F-TTGAGGGAAAAATACAGTCTTGC R-AAGAACTCTCCATCTTCTTTCGTG	2	6	544-675
	C2_At3g17040	Involved in regulating plastidial gene expression and biogenesis	F-TGGGGTTGGATGGAGTGGAAAG R-AGTAGAGGTTACGAATTTCCTCTGC	4	4	400-548
	C2_At2g43360	Catalyzes the conversion of dethiobiotin to biotin	F-TCGATCTCCTCTTTCATGGCG R-TTGAGGACAATACGAACAATCTTC	6	8	943-1067
	C2_At4g38810	Calcium-binding EF hand family protein	F-AGGTGATGGACGGTTCAGATATAATG R-AACTTCTTTAAACTCAGTCTTGCTTAC	1	4	1679-1898
	C2_At4g37280	MRG family protein	F-AAGAAGCAACTGGTTGATGATTGGG R-TCCTTTTTGGATCGGTATTCAAGGTA	2	15	758-968
	Total number of alleles				47	
	Average alleles per locus				7.8	
Tree tomato and relatives	C2_At1g71810	ABC1 family protein	F-TCATGCAGATCCACATCCTGGAAAC R-AGTGACAAAATCCTTGGCCAATGC	4	8	1112-1350
	C2_At4g00090	Transducin family protein / WD-40 repeat family protein	F-AGATATTGGCCACCACTCATGGTTC R-AGGCGACCATGCCATGTCCG	1	8	1450-1740
	C2_At4g32930	Molecular_function unknown	F-TCCTCTTCCTATTGGCAAGGGC R-TGGACACTCCCCCTTTTCATCATAC	11	9	1450-2513
	C2_At3g15430	Regulator of chromosome condensation (RCC1) family protein	F-TCGTTTGAGGTCAACTTTAATGGAGG R-AGTTGTTTCAGGCCCATGACCAAG	7	6	1146-1210
	C2_At1g44760	Universal stress protein (USP) family protein	F-TTCTTCATCTGCTGCTCATCTTGC R-AGAGGGTTTTTTCTGACCCAAGAC	6	8	794-1120
	Total number of alleles				39	
	Average alleles per locus				7.8	

^1^Functional anotation taken from http://arabidopsis.org/. ^2^F = Forward primer. R = Reverse primer. ^3^Location on the tomato genome was taken from (www.sgn.cornell.edu). ^4^PCR size was estimated on agarose gels by using GeneSnap for Windows XP software, as described in material and methods. ^5^bp = base pairs.

**Table 3:** Heterozygosity and fixation indexes per locus and accession.

Species	Average and range of Indexes ^1^					
		H_o_^2^	H_e_^3^	F_IS_^4^	F_IT_^5^	F_ST_^6^
Lulo and relatives	Accession	0.03 (0-0.23)	0.05 (0-0.29)	0.42 (0.4-0.6)	0.96 (0.95-0.97)	0.93 (0.92-0.94)
	Locus	0.03 (0-0.09)	0.05 (0-0.14)	0.47 (0.41-0.50)	0.96 (0.95-0.97)	0.92 (0.9-0.95)
Tree tomato and relatives	Accession	0.003 (0-0.08)	0.003 (0-0.1)	0.86 (0.86-1)	0.99 (0.99-1)	0.99 (0.99-1)
	Locus	0.003 (0-0.01)	0.003 (0-0.02)	0.43 (0.28-1)	0.99 (0.99-1)	0.99 (0.99-1)

^1^Range is indicated in parenthesis, ^2^Observed heterozygosity, ^3^Expected heterozygosity, ^4^Inbreeding coefficient, ^5^Measure of genetic differentiation over populations, ^6^Variance of allele frequencies among populations.
