# Supplementary material for: Genetic variation in the Solanaceae fruit bearing species lulo and tree tomato revealed by Conserved Ortholog (COSII) markers
Source: Genet Mol Biol. 2010 Jun 1;33(2):271–8. doi: 10.1590/S1415-47572010005000016 (PMC3036857; doi:10.1590/S1415-47572010005000016)
Supplement: Table S1 — COSII Markers selected for screening. [file gmb-33-2-271-suppl3.pdf]

Table S1 - COSII Markers selected for screening of lulo and tree tomato entries in the current study. Functional annotation of underlying genes is to be found in http://arabidopsis.org/ or www.sgn.cornell.edu.

Lulo	Tree tomato
C2_At1g02140	C2_At1g10240
C2_At1g16210	C2_At1g16210
C2_At1g50575	C2_At1g26520
C2_At1g56345	C2_At1g44760
C2_At2g04700	C2_At1g50575
C2_At2g06530	C2_At1g71810
C2_At2g18030	C2_At2g06530
C2_At2g18710	C2_At2g34470
C2_At2g20860	C2_At2g34860
C2_At2g34470	C2_At2g39690
C2_At2g34860	C2_At2g43360
C2_At2g39690	C2_At3g04870
C2_At2g43360	C2_At3g10020
C2_At3g01160	C2_At3g10220
C2_At3g04710	C2_At3g11210
C2_At3g06580	C2_At3g15430
C2_At3g09920	C2_At3g16150
C2_At3g10020	C2_At3g17000
C2_At3g10220	C2_At3g17040
C2_At3g11210	C2_At3g19630
C2_At3g12490	C2_At3g21610
C2_At3g16150	C2_At3g28050
C2_At3g17040	C2_At3g28720
C2_At3g19630	C2_At3g52120
C2_At3g21610	C2_At3g58470
C2_At3g25590	C2_At3g61140
C2_At3g28720	C2_At3g62940
C2_At3g46780	C2_At4g00090
C2_At3g52120	C2_At4g03210
C2_At3g58470	C2_At4g12230
C2_At3g61140	C2_At4g14110
C2_At3g62940	C2_At4g22200
C2_At4g03210	C2_At4g24830
C2_At4g22200	C2_At4g32930
C2_At4g24830	C2_At4g35930
C2_At4g34700	C2_At4g37130
C2_At4g37130	C2_At4g37280
C2_At4g37280	C2_At5g20180
C2_At4g38810	C2_At5g23880
C2_At5g06430	C2_At5g62390
C2_At5g20180	C2_At5g66530
C2_At5g23880	
C2_At5g46630	
C2_At5g62390	
C2_At5g62440	
C2_At5g66530	
