# Supplementary material for: Genetic variation in the Solanaceae fruit bearing species lulo and tree tomato revealed by Conserved Ortholog (COSII) markers
Source: Genet Mol Biol. 2010 Jun 1;33(2):271–8. doi: 10.1590/S1415-47572010005000016 (PMC3036857; doi:10.1590/S1415-47572010005000016)
Supplement: Table S3 — Genetic distances for tree tomato and relatives. [file gmb-33-2-271-suppl5.pdf]

Table S3 - Genetic distance estimates for tree tomatoes and wild relatives used to construct the UPGMA tree in Figure S2.

Acce*	01	04	07	08	09	10	11	13	14	15	16	18	20	22	23	24	25	26	28	31	32	33	34	35	39	41	42	45	47	50
01		0.8	0.4	0.8	1	0.6	1	1	1	1	1	1	0.8	1	1	1	1	1	1	1	1	1	0.6	0.4	0.8	0.8	0.75	1	1	0.8
04			0.8	0.4	0.8	0.6	0.8	0.6	1	0.6	1	1	0.6	1	1	1	1	1	1	1	1	1	0.6	0.8	0.6	0.6	0.85	0.8	0.8	0.8
07				0.6	0.8	0.4	0.6	1	1	0.8	0.6	0.6	0.8	0.6	0.8	1	0.8	0.6	0.6	0.8	0.8	0.8	0.4	0.6	0.8	1	0.75	1	1	0.8
08					0.4	0.2	0.6	0.4	0.8	0.2	0.8	0.8	0.6	0.8	0.6	1	0.8	0.6	0.8	1	0.8	0.8	0.4	0.8	0.4	0.6	0.85	0.8	0.8	0.6
09						0.4	0.8	0.6	0.6	0.4	0.8	0.6	0.8	0.8	0.4	1	0.6	0.4	0.6	1	0.6	0.6	0.8	1	0.6	0.8	0.85	1	0.6	0.4
10							0.8	0.6	0.8	0.4	0.8	0.8	0.6	0.8	0.6	1	0.8	0.6	0.8	1	0.8	0.8	0.6	0.8	0.4	0.8	0.65	1	0.8	0.4
11								0.6	1	0.4	0.6	0.4	1	0.6	0.8	1	0.8	0.6	0.6	0.8	0.8	0.8	0.6	1	1	0.8	0.95	0.8	0.8	1
13									0.8	0.2	1	1	0.8	1	0.8	0.8	1	0.8	1	0.8	1	1	0.8	1	0.6	0.6	0.85	0.8	0.6	0.6
14										0.8	1	0.6	0.8	0.8	0.6	0.8	0.4	0.6	0.6	1	0.8	0.6	1	1	0.8	1	0.95	0.8	1	0.6
15											0.8	0.8	0.8	0.8	0.6	1	0.8	0.6	0.8	1	0.8	0.8	0.6	1	0.6	0.6	0.85	0.8	0.6	0.6
16												0.6	1	0.4	0.6	1	0.8	0.6	0.6	0.4	0.6	0.6	0.8	1	0.8	1	0.95	1	1	1
18													0.8	0.4	0.6	0.8	0.4	0.4	0.2	0.8	0.6	0.4	0.8	1	1	1	0.95	1	1	0.8
20														0.8	1	0.8	0.8	1	0.8	1	1	0.8	0.8	0.8	0.6	0.8	0.85	1	0.6	0.8
22															0.8	0.8	0.6	0.6	0.2	0.8	0.6	0.6	0.8	1	1	0.8	0.95	0.8	0.8	1
23																1	0.6	0.4	0.6	0.8	0.2	0.6	0.8	1	0.6	1	0.95	1	1	0.6
24																	0.6	1	0.8	0.6	1	0.8	1	1	1	1	0.95	1	1	1
25																		0.6	0.4	1	0.6	0.4	0.8	1	1	1	0.95	0.8	1	0.8
26																			0.4	0.8	0.6	0.6	0.8	0.8	0.8	1	0.95	1	1	0.6
28																				0.8	0.4	0.4	0.8	1	1	0.8	0.95	0.8	0.8	0.8
31																					0.8	0.8	1	1	0.8	1	0.95	1	1	1
32																						0.6	0.8	1	0.8	0.8	0.95	0.8	0.8	0.8
33																							0.6	1	1	1	0.95	1	1	0.8
34																								0.6	0.8	0.8	0.95	0.8	1	1
35																									0.6	1	0.75	1	1	1
39																										0.8	0.65	1	0.8	0.6
41																											0.85	0.6	0.6	0.8
42																												0.95	0.85	0.65
45																													0.8	0.8
47																														0.8
50																														

*Accessions. All accessions are represented by their last two numbers.
