# Supplementary material for: Genetic variation in the Solanaceae fruit bearing species lulo and tree tomato revealed by Conserved Ortholog (COSII) markers
Source: Genet Mol Biol. 2010 Jun 1;33(2):271–8. doi: 10.1590/S1415-47572010005000016 (PMC3036857; doi:10.1590/S1415-47572010005000016)
Supplement: Figure S2 — Consensus tree using bootstrapping of 10,000 replicates for tree tomato. [file gmb-33-2-271-suppl2.pdf]

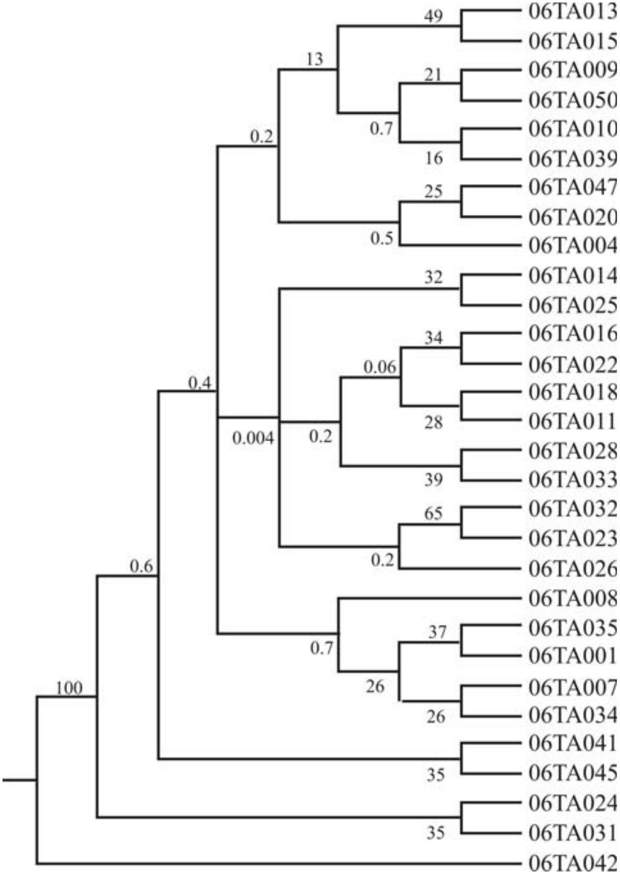

Figure S2 - Consensus tree using bootstrapping of 10,000 replicates for tree tomato.
